# Impact of Foliar Application of ZnO and Fe_3_O_4_ Nanoparticles on Seed Yield and Physio-Biochemical Parameters of Cucumber (*Cucumis sativus* L.) Seed under Open Field and Protected Environment *vis a vis* during Seed Germination

**DOI:** 10.3390/plants11233211

**Published:** 2022-11-23

**Authors:** Nakul Gupta, Sudhir Kumar Jain, Bhoopal Singh Tomar, Anjali Anand, Jogendra Singh, Vidya Sagar, Rajesh Kumar, Vikas Singh, Tribhuvan Chaubey, Kamel A. Abd-Elsalam, Awani Kumar Singh

**Affiliations:** 1 ICAR—Indian Institute of Vegetable Research, PB-01, Po-Jakhini (Sahanshahpur), Varanasi 221305, India; 2 ICAR—Indian Agricultural Research Institute, New Delhi 110012, India; 3 Plant Pathology Research Institute, Agricultural Research Center, Giza 12619, Egypt

**Keywords:** cucumber, foliar application, Zn-nanoparticles, Fe-nanoparticles, seed germination, antioxidant

## Abstract

Nutritionally rich cucumber seeds remain in demand in the agricultural, health and cosmetic sectors as they are essential for a successful crop stand establishment and seed-based products. However, the production of cucumber seeds is impeded by source limitation and nutrient deficiency. The foliar application of micronutrients can supplement this deficiency and overcome the physiological setback. An experiment was undertaken to compare the impacts of the foliar application of Fe and Zn, as nanoparticles and fertilizers, on the yield and seed quality of cucumber under open and protected environments. A foliar spray of nano-ZnO (ZnNPs) and nano-Fe_3_O_4_ (FeNPs) at 100, 200 and 300 mg L^−1^, as well as ZnSO_4_ and FeSO_4_ as fertilizer (0.5%), was conducted at the vegetative stage and pre- and post-flowering stages. The NPs had a greater efficacy in an open field than in the protected (naturally ventilated poly house) environment. The application of both NPs increased seed yield (51.7–52.2%), total chlorophyll content (15.9–17.3%) and concentration of Zn and Fe in the fruit and the seed, by 2.0–58.5% and 5.0–30.5%, respectively. A significant increase in starch, soluble proteins, soluble sugars and oil content was observed in the seeds from the NP treated plants. NP treatment also enhanced the germination-related parameters, such as percent germination (16.8–17.0%), rate of germination (18.0–22.2%) and seedling vigor (59.8–72.6%). The biochemical characterization showed a significant improvement in the seed water uptake and the activity of hydrolytic enzymes (amylase and protease) in the germinating seed. The involvement of reactive oxygen species (superoxide anion and hydrogen peroxide) and antioxidant enzymes (Superoxide dismutase, Catalase and Peroxidase) in the germination process was indicated by an increase in their activities in the seeds from NP treated plants. Hence, the study proposes the potential benefit of the foliar application of 300 mg L^−1^ ZnNPs and 200 mg L^−1^ FeNPs at crucial stages of plant growth to improve the yield and seed quality in cucumbers.

## 1. Introduction

Cucumber (*Cucumis sativus* L.) is an agriculturally important low-calorie vegetable and is consumed as a salad. Its seeds are nutrient-dense and contain phytonutrients, fibre and antioxidants that help in immunity, skin smoothening, weight loss, eye care and the prevention of cancer [1]. However, seed production in cucumbers is inefficient due to a higher production of underdeveloped seeds with poor quality [2,3,4]. Amongst the various constraints, improper pollination, source-sink limitation [2,5] and nutrient deficiency [6,7] have been attributed to the formation of immature and inferior quality seeds. The foliar application of micronutrients that are crucial for plant growth, seed development and germination, such as iron (Fe) and zinc (Zn), has been proven to resolve this issue in many vegetable crops, for example, in wheat, rice, maize and Arabidopsis [8,9,10,11]. Furthermore, the deficiency of both of these micronutrients is very common in various crops and Indian soils [12,13]; moreover, Zn- and Fe-based micro-nutrient malnutrition affects over three billion people worldwide [14]. These micronutrients render a more meaningful solution when sprayed in the form of nanoparticles as it enables a controlled and targeted delivery due to its application in minute quantity. It also reduces phytotoxicity, soil pollution and other environmental threats to the food chain [15,16].

The physiochemical properties of NPs show that they are less than 100 nm in size and possess a high surface area, reactivity, solubility, penetration capacity, and surface/volume ratio, deeming them environmentally safe for application in agricultural crops compared to their counterparts of higher concentration foliar sprays [17,18]. Numerous researchers have documented the positive effects of Zn- and Fe-NPs on yield and quality in barley [19], maize [20], sunflower [21], wheat [22] and peach [23]. However, the effect of foliar spray of NPs on the nutritional quality of seeds in terms of the seed reserves and micronutrient content has not been investigated. Information on the physio-biochemical changes that take place during germination in the improved seed from NP treated plants is also lacking. The ROS and antioxidants play an important role in plant signaling networks related to seed germination, dormancy, seedling establishment, plant growth, plant stress responses, cell division and cell death [24]. The activity of dehydrogenase and other hydrolytic enzymes, e.g., amylase and protease, are linked to the germination ability of the seeds. High vigor seeds show a higher activity of both the enzymes during germination [25]. With the exception of foliar application of NPs, micronutrients are generally sprayed in the form of solutions with a higher concentration, but a comparison of the use of both types for seed quality improvement has not been studied. Therefore, the present investigation was aimed to understand the impact of the foliar application of nano-ZnO (ZnNPs) and nano-Fe_3_O_4_ (FeNPs), *vis-a-vis* solutions of their higher concentrations, on (i) seed quality and yield components (ii) seed reserve and micronutrient accumulation (iii) physiological and biochemical changes related to germination in the seeds.

## 2. Results

### 2.1. Characterization of ZnO and Fe_3_O_4_ Nano-Particles

**Figure 1:**
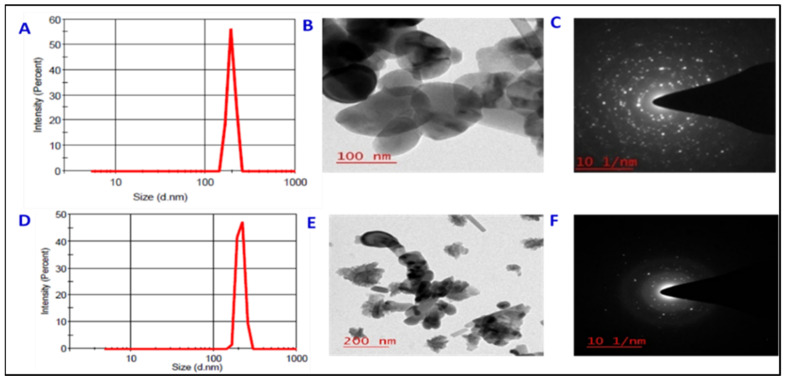
(**A**) DLS size distribution of ZnO-NPs, (**B**) TEM image of ZnO-NPs, (**C**) SAED pattern of the ZnO-nanocrystals, (**D**) DLS size distribution of Fe_3_O_4_-NPs, (**E**) TEM image of Fe_3_O_4_-NPs and (**F**) SAED pattern of the Fe_3_O_4_-nanocrystals.

The purity of synthesized ZnO-NPs and Fe_3_O_4_-NPs was 98.3% and 97.0%, respectively. Dynamic light scattering (DLS) measurements showed that ZnO-NPs and Fe_3_O_4_-NPs had an average hydrodynamic diameter of ~193 nm and ~210 nm, respectively, which were positively correlated with their primary sizes (Figure 1); meanwhile, the surface area of ZnO-NPs and Fe_3_O_4_-NPs was around 20 m^2^g^−1^ and 25 m^2^g^−1^, respectively, which was in good agreement with the enhancement in sorption capacity of nanoparticles. The transmission electron microscopy (TEM) images showed that the ZnO-NPs were spherical with an average size of ~70 nm, whereas the Fe_3_O_4_-NPs had an average primary size of ~55 nm and were quasi-spherical in shape (Figure 1B,E). These are consistent with our characterization results of both the NPs from the same batch [26]. The zeta potential of the ZnNPs and FeNPs was −26.20 ± 1.08 mV and −29.50 ± 2.02 mV, respectively. The typical selected area of electron diffraction pattern (SAED) in planes (1 0 1), (1 0 2), (1 1 0), (0 0 2), (1 0 0), (1 0 3) of the ZnO-NPs with eleven sharp and bright concentric rings, confirm hexagonal structure and crystalline nature. However, a selected area of the electron diffraction pattern (Figure 1C,F)) consisted of diffraction spots/rings that were indexed with a correspondence to the magnetite spinal structure (4 4 0), (5 1 1), (4 2 2), (4 0 0), (3 1 1) and (2 2 0) planes, which is characteristic of the diffraction ring pattern of polycrystalline face centered cubic (FCC) crystal structure of Fe_3_O_4_.

### 2.2. Physio-Chemical Properties of Soil

Soil properties and micronutrient (Fe and Zn) content under open and protected environments are listed in Table 1.

### 2.3. Plant Growth and Total Chlorophyll Content

Irrespective of varieties, seasons and environment, the foliar spray of ZnNPs (300 mg L^−1^) and FeNPs (200 mg L^−1^) increased the plant growth, measured in terms of vine length and number of fruits per vine and fruit attributes, such as fruit dimension and weight (Table 2). The improvement in the growth parameters was significantly higher in micronutrient supplied through NPs over their bulk counterparts. Furthermore, the percentage of increase in plant growth with the foliar application of NPs over the control was higher in the open field environment than in the protected environment. The percent increase in total chlorophyll content from middle leaves was greater in plants treated with 300 mg L^−1^ ZnNPs (17.5% and 17.2%), followed by 200 mg L^−1^ FeNPs (16.3% and 15.9%), under both open field and protected environment, respectively (Table 3).

### 2.4. Seed Yield and Quality Attributes

The foliar application of NPs and other treatments had no significant effect on seed dimensions (length and breadth) under both the environments (Supplementary Table S1). The other seed yield parameters, such as1000-seed weight, number of filled seed and seed yield per hectare, were significantly increased with the foliar application of NPs, irrespective of varieties, seasons and environments. The largest increase in the traits of 1000-seed weight, number of filled seed and seed yield per plant were registered in plants treated with 300 mg L^−1^ ZnNPs (5.2, 84.1 and 52.2%, respectively), followed by 200 mg L^−1^ FeNPs (4.9, 82.2 and 51.7%, respectively) (Table 2).

The seed quality, measured in terms of seed germination and vigor indices, was largest in the seeds obtained from plants treated with 200 mg L^−1^ FeNPs and 300 mg L^−1^ ZnNPs over other treatments under both the environment (Table 3). The increase in the seed vigor indices was a result of the significant increase in seedling length and seedling dry weight, although a higher concentration of FeNPs, at 300 mg L^−1^, showed a significant reduction in seed quality. Germination matrix data showed a significant difference in speed of germination, area under curve (AUC), mean germination time and t50 germination (time taken for 50% germination) in NPs-treated seeds over untreated control (Supplementary Figure S1).

### 2.5. Seed Chemical Composition

Increased TSS, TSP, starch and oil content in NPs-treated seeds resulted in the development of bolder seeds under both the environments. The percentage of increase with NPs application in all of the compositional parameters, with the exception of oil content, was higher in the open field than in the protected environment. Zn supplication as NPs (300 mg L^−1^) increased TSS, TSP, starch and oil content up to 14.9, 24.3, 45.2 and 21.4%, respectively, over the untreated treatment. The application of Fe as NPs (200 mg L^−1^) increased TSS, TSP, starch and oil content up to 13.8, 19.7, 43.5 and 17.5%, respectively, over the untreated treatment (Table 4).

**Table 2:** Effects of foliar application of Zn and Fe (NPs and bulk) on plant growth, fruits and seed yield attributes in cucumber under open field (E1) and protected environment (E2).

Treatment	Vine Length (cm)		Number of Fruits per Vine		Fruit Weight (g)		Fruit Length (cm)		Fruit Width (cm)		Total Number of Seeds per Fruit		1000-Seed Weight (g)		Seed Yield (kg/ha)	
	E1	E2	E1	E2	E1	E2	E1	E2	E1	E2	E1	E2	E1	E2	E1	E2
100 mg L^−1^ ZnNPs	168.8 ± 4.2 bc	269.0 ± 4.8 bc	1.84 ± 0.01 b	2.64 ± 0.02 b	390.0 ± 12.5 ab	517 ± 14.2 ab	20.48 ± 0.81 bc	24.31 ± 0.52 bc	6.96 ± 0.05 b	7.91 ± 0.07 ab	371.0 ± 10.1 bc	386.5 ± 12.5 bc	24.55 ± 0.21 b	24.93 ± 0.18 bc	241.0 ± 14.2 b	353.4 ± 12.5 bc
200 mg L^−1^ ZnNPs	178.5 ± 4.7 b	281.5 ± 5.2 b	1.91 ± 0.01 b	2.73 ± 0.02 b	397.0 ± 11.1 ab	520.5 ± 17.4 ab	21.53 ± 0.74 b	25.26 ± 0.88 ab	7.33 ± 0.06 ab	8.07 ± 0.04 ab	384.0 ± 11.6 bc	397.3 ± 13.4 ab	25.05 ± 0.15 a	25.17 ± 0.31 b	264.8 ± 11.5 b	377.5 ± 11.9 ab
300 mg L^−1^ ZnNPs	204.3 ± 3.9 a	308.0 ± 5.0 ab	2.10 ± 0.03 ab	3.15 ± 0.03 a	418.0 ± 10.3 a	562.8 ± 10.8 a	23.23 ± 0.66 a	26.61 ± 0.90 a	7.55 ± 0.03 a	8.36 ± 0.03 a	413.5 ± 16.2 a	428.3 ± 11.4 a	25.32 ± 0.35 a	25.72 ± 0.30 a	295.6 ± 12.4 a	411.1 ± 10.6 a
100 mg L^−1^ FeNPs	193.0 ± 5.1 ab	283.8 ± 4.1 b	1.84 ± 0.02 b	2.88 ± 0.02 b	408.0 ± 14.5 ab	541.5 ± 11.6 ab	21.63 ± 0.69 b	25.61± 0.51 ab	7.06 ± 0.02 ab	8.13 ± 0.03 ab	383.0 ± 12.2 bc	393.8 ± 14.5 b	24.53 ± 0.28 bc	25.08 ± 0.28 b	249.7 ± 12.7 b	367.8 ± 17.5 b
200 mg L^−1^ FeNPs	211.0 ± 5.3 a	317.3 ± 3.3 a	2.17 ± 0.02 a	3.17 ± 0.03 a	421.5 ± 9.6 a	565.3 ± 12.4 a	23.28 ± 0.80 a	26.60 ± 0.46 a	7.60 ± 0.02 a	8.45 ± 0.06 a	409.5 ± 13.4 a	423.5 ± 9.9 a	25.24 ± 0.22 a	25.65 ± 0.11 a	294.6 ± 13.3 a	406.0 ± 16.4 a
300 mg L^−1^ FeNPs	180.0 ± 3.6 ab	296.0 ± 4.7 ab	1.91 ± 0.01 b	2.99 ± 0.01 ab	397.3 ± 15.1 ab	540.3 ± 13.4 ab	21.78 ± 0.73 b	23.41 ± 0.62 c	6.30 ± 0.03 c	7.72 ± 0.02 b	394.5 ± 11.4 ab	407.5 ± 10.6 ab	24.64 ± 0.19 b	24.88 ± 0.14 bc	239.9 ± 10.9 b	331.2 ± 12.4 c
0.5% ZnSO_4_	163.3 ± 4.5 bc	249.8 ± 3.4 c	1.30 ± 0.01 c	2.36 ± 0.02 c	364.8 ± 12.8 b	485.3 ± 10.9 b	20.13 ± 0.41 c	24.76 ± 0.68 b	6.26 ± 0.05 c	7.66 ± 0.02 b	368.0 ± 12.8 bc	381.3 ± 13.5 bc	24.32 ± 0.26 bc	24.79 ± 0.20 c	204.2 ± 11.8 c	305.4 ± 13.4 cd
0.5% FeSO_4_	157.3 ± 4.0 bc	238.8 ± 4.5 c	1.22 ± 0.02 c	2.32 ± 0.03 c	358.8 ± 10.5 b	480.8 ± 11.5 b	19.98 ± 0.59 cd	24.11 ± 0.71 bc	6.29 ± 0.03 c	7.72 ± 0.04 b	365.0 ± 9.8 c	378.3 ± 12.7 bc	24.23 ± 0.23 c	24.75 ± 0.22 c	203.3 ± 12.4 c	303.1 ± 12.4 cd
Control	150.0 ± 3.8 c	228.0 ± 4.4 c	1.16 ± 0.01 c	2.25 ± 0.03 c	348.5 ± 11.4 b	456.5 ± 13.7 b	18.88 ± 0.62 d	22.26 ± 0.70 c	6.02 ± 0.02 c	7.01 ± 0.05 c	352.0 ± 10.2 c	365.3 ± 13.7 c	24.06 ± 0.17 c	24.62 ± 0.19 c	194.3 ± 13.1 c	278.9 ± 13.1 d

Values in the table are mean of two varieties from two seasons using three replications ± standard error (SE). Mean followed by the same scripts (a, b, c, ab, etc.) are not significantly different (*p* < 0.05).

**Table 3:** Effects of foliar application of Zn and Fe (NPs and bulk) on total leaf chlorophyll and seed quality attributes in cucumber under open field (E1) and protected environment (E2).

Treatment	Total Chlorophyll from Leaf (mg g^−1^ FW)		Seed Germination (%)		Seed Vigour Index-I		Seed Vigour Index-II	
	E1	E2	E1	E2	E1	E1	E1	E2
100 mg L^−1^ ZnNPs	20.3 ± 0.94 cd	21.7 ± 0.83 c	75.40 ± 2.17 b	78.90 ± 3.15 b	1301.0 ± 20.2 c	1543.8 ± 17.6 c	11.73 ± 0.11 c	12.82 ± 0.18 c
200 mg L^−1^ ZnNPs	22.7 ± 0.88 b	23.5 ± 0.92 b	77.65 ± 2.25 b	80.40 ± 2.85 b	1567.2 ± 23.5 b	1796.3 ± 26.2 b	12.66 ± 0.13 bc	13.42 ± 0.08 c
300 mg L^−1^ ZnNPs	25.4 ± 1.01 a	26.7 ± 0.98 a	81.79 ± 2.10 a	85.13 ± 2.55 a	1807.6 ± 26.6 a	2061.3 ± 29.6 a	14.95 ± 0.14 a	16.16 ± 0.11 a
100 mg L^−1^ FeNPs	23.1 ± 0.90 b	22.9 ± 0.81 bc	76.52 ± 1.78 b	79.79 ± 3.05 b	1479.4 ± 19.5 b	1728.8 ± 16.5 b	13.26 ± 0.09 b	14.40 ± 0.09 b
200 mg L^−1^ FeNPs	25.2 ± 0.78 a	26.1 ± 0.91 a	81.90 ± 2.13 a	85.4 ± 2.41 a	1780.1 ± 30.1 a	2011.1 ± 21.5 a	14.72 ± 0.10 a	15.95 ± 0.15 a
300 mg L^−1^ FeNPs	21.7 ± 0.91 bc	23.4 ± 0.90 b	72.65 ± 1.89 c	77.63 ± 3.10 b	1312.2 ± 17.5 c	1569.4 ± 18.4 c	10.43 ± 0.12 d	11.74 ± 0.12 d
0.5% ZnSO_4_	19.1 ± 0.99 d	21.8 ± 0.89 c	73.75 ± 2.31 c	77.00 ± 2.88 b	1263.7 ± 24.2 c	1480.0 ± 16.9 c	10.51 ± 0.13 d	11.59 ± 0.13 d
0.5% FeSO_4_	18.7 ± 0.86 d	21.2 ± 0.96 c	74.13 ± 1.94 c	76.86 ± 1.85 b	1273.0 ± 19.6 c	1479.9 ± 23.3 c	9.82 ± 0.10 e	10.72 ± 0.10 d
Control	16.9 ± 0.95 e	19.3 ± 0.87 d	70.00 ± 2.01 d	73.15 ± 2.05 c	1114.3 ± 18.4 d	1323.0 ± 20.4 d	8.66 ± 0.11 f	9.55 ± 0.14 e

Values in the table are mean of two varieties from two seasons using three replications ± standard error (SE). Mean followed by the same scripts (a, b, c, ab, etc.) are not significantly different (*p* < 0.05).

**Table 4:** Effects of foliar application of Zn and Fe (NPs and bulk) on seed compositional parameters in cucumber under open field (E1) and protected environment (E2).

Treatment	Total Soluble Sugars (mg g^−1^ DW)		Total Soluble Proteins (mg g^−1^ DW)		Total Starch (mg g^−1^ DW)		Oil Content (%)	
	E1	E2	E1	E2	E1	E2	E1	E2
100 mg L^−1^ ZnNPs	7.129 ± 0.36 c	7.279 ± 0.17 c	171.8 ± 13.2 b	197.4 ± 12.3 bc	142.4 ± 9.8 cd	152.8 ± 12.4 c	30.29 ± 0.25 ab	30.97 ± 0.19 b
200 mg L^−1^ ZnNPs	7.342 ± 0.27 b	7.443 ± 0.28 b	180.8 ± 12.5 ab	208.2 ± 14.6 ab	154.2 ± 11.5 b	165.1 ± 14.7 b	30.43 ± 0.31 a	31.22 ± 0.22 a
300 mg L^−1^ ZnNPs	7.573 ± 0.41 a	7.692 ± 0.38 a	194.1 ± 17.5 a	219.5 ± 22.5 a	172.9 ± 10.2 a	184.9 ± 16.4 a	30.49 ± 0.19 a	31.43 ± 0.31 a
100 mg L^−1^ FeNPs	7.221 ± 0.32 bc	7.332 ± 0.33 b	174.3 ± 9.5 b	200.4 ± 20.1 b	144.3 ± 13.5 c	157.0 ± 10.6 b	30.11 ± 0.21 b	31.07 ± 0.27 ab
200 mg L^−1^ FeNPs	7.503 ± 0.19 a	7.781 ± 0.25 a	186.9 ± 11.3 a	216.7 ± 11.8 a	170.9 ± 12.6 a	180.5 ± 14.3 a	30.38 ± 0.18 a	31.31 ± 0.11 a
300 mg L^−1^ FeNPs	7.089 ± 0.22 d	7.248 ± 0.19 c	172.8 ± 14.2 b	196.5 ± 15.6 bc	133.4 ± 13.9 d	135.7 ± 11.2 d	29.99 ± 0.26 c	30.98 ± 0.21 b
0.5% ZnSO_4_	7.116 ± 0.29 c	7.389 ± 0.22 b	168.6 ± 11.5 bc	198.8 ± 16.2 bc	135.3 ± 8.6 d	140.6 ± 13.4 d	30.31 ± 0.20 a	30.93 ± 0.26 b
0.5% FeSO_4_	7.032 ± 0.18 d	7.325 ± 0.39 b	169.6 ± 10.5 bc	195.3 ± 19.8 bc	140.7 ± 9.5 cd	146.3 ± 10.7 cd	30.23 ± 0.18 b	30.89 ± 0.18 b
Control	6.593 ± 0.21 e	7.138 ± 0.30 d	156.1 ± 16.7 c	189.6 ± 14.8 c	119.1 ± 11.2 e	129.5 ± 6.5 d	29.87 ± 0.16 c	30.77 ± 0.23 c

Values in the table are mean of two varieties from two seasons using three replications ± standard error (SE). Mean followed by the same scripts (a, b, c, ab, etc.) are not significantly different (*p* < 0.05).

### 2.6. Electrical Conductance (EC) and Dehydrogenases Activity from Seeds

**Figure 2:**
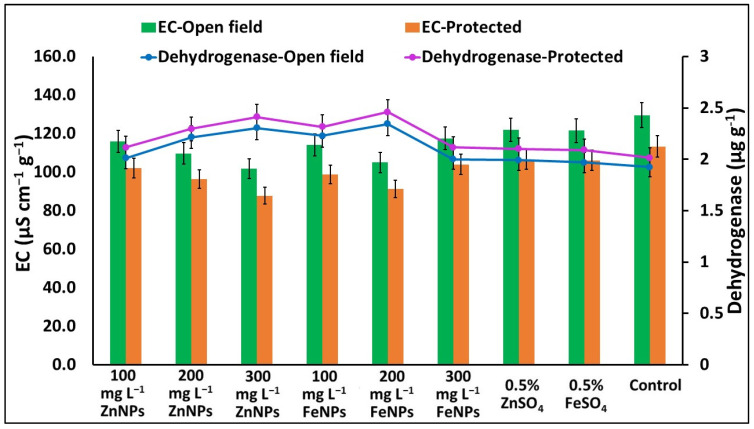
Effects of foliar application of Zn & Fe (nano & bulk) particles on electrical conductivity and dehydrogenases enzyme activity from seed leachates in different environments. Data represent the mean values ± SE of two varieties from two seasons using three replicates and different letters in the figure indicate significant differences among different treatments at the *p <* 0.05 level.

Irrespective of varieties and seasons, the minimal values for EC (101.8 and 87.8 µS cm^–1^ g^–1^) were registered as 300 mg L^−1^ ZnNPs, followed by 200 mg L^−1^ FeNPs (105.1 and 91.2 µS cm^–1^ g^–1^), in both the open field and protected environment, respectively. However, the maximal EC values (129.6 and 113.4 µS cm^–1^ g^–1^) were recorded for the untreated control in the open field and protected environment, respectively (Figure 2). The dehydrogenases activity in the seeds from the NPs-treated plants significantly differed to the un-treated control in both the varieties and in both the studied environments. The dehydrogenases activities in the seeds were 1.22- & 1.25-folds higher with 200 mg L^−1^ FeNPs-treated plants, followed by 1.20- & 1.23-folds higher with 300 mg L^−1^ ZnNPs-treated plants, over the untreated control in the open field and protected environment, respectively (Figure 2).

### 2.7. Water Uptake and Hydrolytic Enzyme Activity in Germinating Seeds

**Figure 3:**
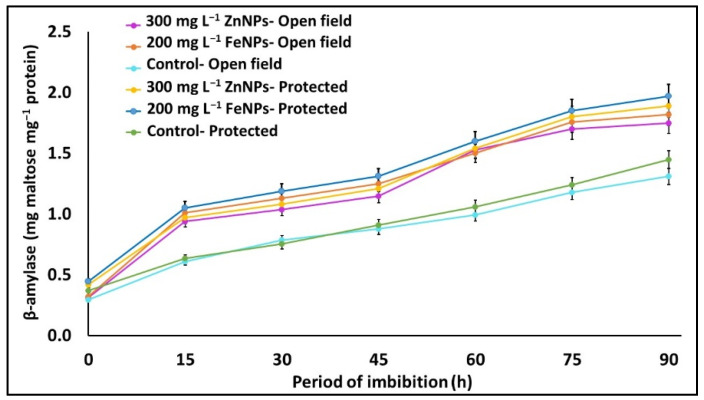

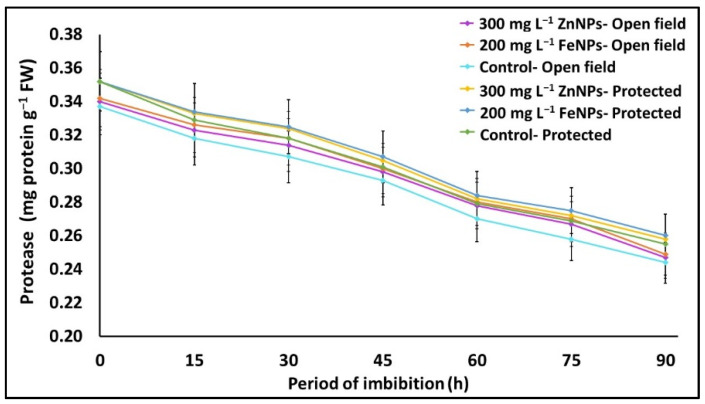
Effects of foliar application of Zn & Fe (nano & bulk) particles on water uptake and hydrolytic enzyme activity (β-amylase and protease enzyme) in germinating seeds in different environments. Data represent the mean values ± SE of two varieties from two seasons using three replicates and different letters in the figure indicate significant differences among different treatments at the *p <* 0.05 level.

Figure 3 shows that the seeds from the NPs-treated plants recorded an increased and rapid water uptake compared to the untreated control during seed germination. The tri-phasic water uptake curve of seed germination showed a shortening of phase II (6–10 h) and an early start of phase III (52–54 h) compared to those of the untreated control (62–66 h). During the seed germination process, from 0 to 90 h, a higher activity of hydrolytic enzymes (β-amylase and protease) was observed in the NPs-treated seeds compared to the untreated control. The β-amylase activity, during seed germination (0–90 h), increased 5.6 and 4.5-folds with mg L^−1^ ZnNPs, and 5.6 and 4.4-folds with 200 mg L^−1^ FeNPs, in the open field and protected environment, respectively. The protease activity estimated, as decline in protein contents per gram of seeds, was also greater in the 300 mg L^−1^ ZnNPs and the 200 mg L^−1^ FeNPs treatment (Figure 3).

### 2.8. Micronutrient (Fe and Zn) Content in Fruits and Seeds

**Figure 4:**
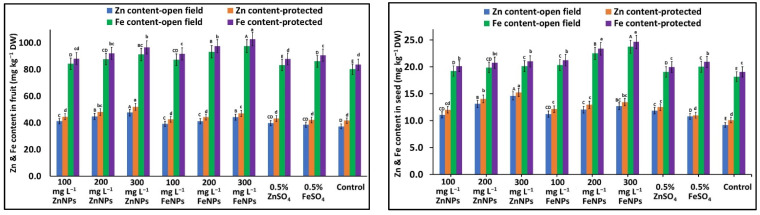
Effects of foliar application of Zn & Fe (nano & bulk) particles on translocation of Zn and Fe contents in fruit and seeds in cucumber grown under different environments. Data represent the mean values ± SE of two varieties from two seasons using three replicates and different letters in the figure indicate significant differences among different treatments at the *p <* 0.05 level.

The Zn and Fe content in fruits and seeds significantly differed in treated plants under both the environments. Fruits from the NP-treated plants showed an increase in Zn content (5.0–27.4% and 2.0–24.6%), whereas fruits from the bulk micronutrient-treated plants registered (3.3–6.6% and 5.2–8.6%) in the open field and protected environment, respectively. Similarly, seeds from the NPs-treated plants registered an increase in Zn content (14.2–58.5% and 12.7–50.8%), whereas seeds from the bulk micronutrient-treated plants registered an increase (17.0–28.9 and 9.0–23.8%) in the open field and protected environment, respectively (Figure 4). Similarly, the fruits from the NP-treated plants recorded an increase in Fe contents (5.0–21.5% and 5.5–23.0%), whereas fruits from the bulk fertilizer-treated plants registered an increase (3.9–7.4% and 5.2–8.6%) in the open field and protected environment, respectively. Moreover, seeds from the NPs-treated plants showed increased Fe content (5.8–30.6% and 5.5–29.2%); whereas seeds from the bulk fertilizer-treated plants increased (5.1–10.2% and 4.8–9.7%) in the open field and protected environment, respectively (Figure 4).

### 2.9. ROS and Antioxidant Enzyme System in Seedling

**Figure 5:**
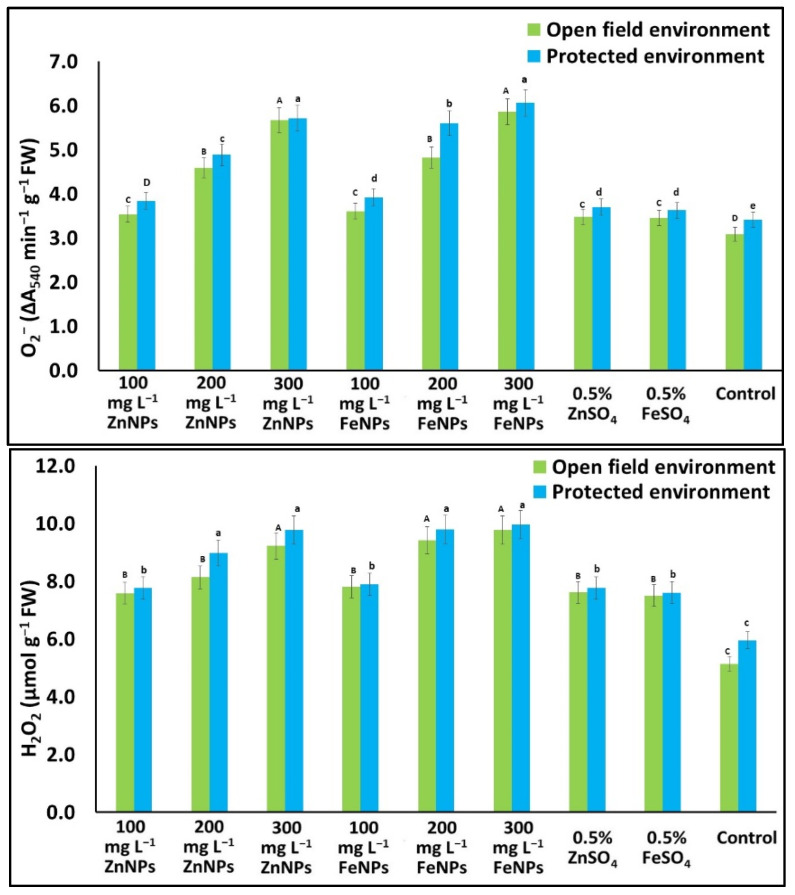
Effects of foliar application of Zn & Fe (nano & bulk) particles on ROS activity (O^2-.^ and H_2_O_2_) in cucumber seed grown under different environments. Data represent the mean values ± SE of two varieties from two seasons using three replicates and different letters in the figure indicate significant differences among different treatments at the *p <* 0.05 level.

The ROS and antioxidant enzyme activity in the seeds from NPs- and bulk- (non-NPs) treated plants were higher than in the control; the increase was directly proportional to the increase in doses of NPs, regardless of growing environments. H_2_O_2_ in the seeds from NPs-treated (100 to 300 mg L^−1^) plants increased (47.3 to 89.8% & 30.3 to 67.2%), whereas in the bulk-treated plants it increased (45.7 to 48.0% & 27.6 to 30.4%) over the control, in the open field and protected environment, respectively. Similarly, O_2_^−^ in the seeds from the NPs-treated plants increased (14.4 to 90.0% & 12.6 to 77.7%), whereas in the bulk (non-NPs)-treated plants it increased (12.1 to 12.6% & 6.3 to 8.5%) over the control, in the open field and protected environment, respectively (Figure 5).

**Figure 6:**
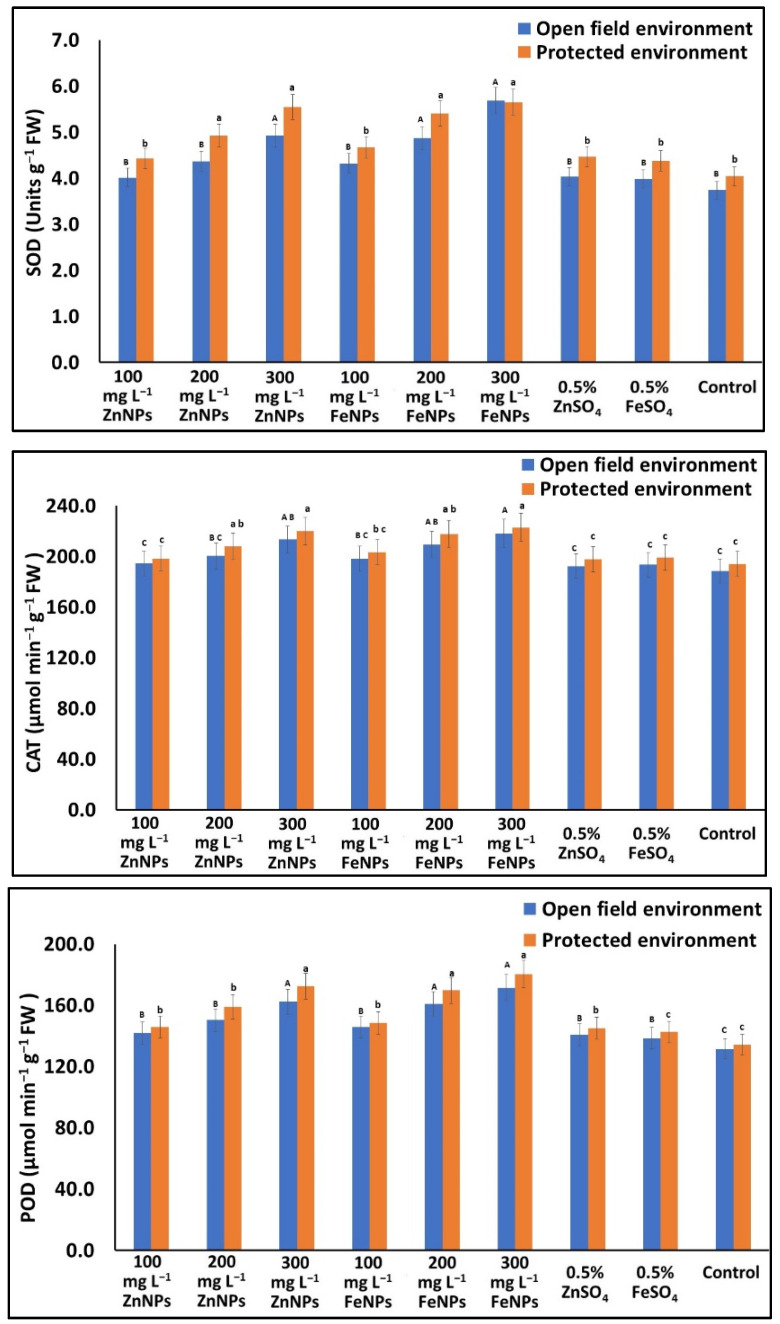
Effects of foliar application of Zn & Fe (nano & bulk) particles on antioxidant enzyme activity in cucumber seed grown under different environments. Data represent the mean values ± SE of two varieties from two seasons using three replicates and different letters in the figure indicate significant differences among different treatments at the *p <* 0.05 level.

Regardless of varieties and seasons, the SOD activity in the seeds from the NPs-treated (100 to 300 mg L^−1^) plants increased (7.4 to 52.1% & 9.3 to 39.5%), whereas in the bulk (non-NPs)-treated plants it increased (6.5 to 7.8% & 8.2 to 10.4%) over the control, in the open field and protected environment, respectively (Figure 6). Similarly, to SOD, CAT & POD activities in the seeds from the NPs-treated plants increased (3.0 to 15.8% & 2.2 to 17.7%; and 7.9 to 30.4% & 8.6 to 34.4%), whereas from the bulk (non-NPs)-treated plants it increased (2.0 to 2.5% & 1.8 to 2.0%; and 5.3 to 6.9% & 6.1 to 7.9%) over the untreated control, in the open field and protected environment, respectively (Figure 6).

## 3. Discussion

A higher percentage of unfilled or underdeveloped seeds in cucumber is a consequence of improper pollination [27,28,29], over-supply of ovule, which limits pollen availability [30] and source limitation to fill the pollinated seeds [2,5]. Source limitation may be resolved by managing plant resources, either through regulating fruit load [2] or by exogenous application of micronutrients and/or plant growth regulators, to ensure an adequate supply to the developing seed. Foliar application of micronutrient in nano-form is being practiced to improve plant growth and seed quality by modulating plants’ physiological response [31,32]. In the present study, pollination was ensured manually under both the growing environments, and the micronutrients (Zn and Fe) were applied as NPs or bulk, through foliar fertigation onto cucumber plants [9,33] to investigate their physio-biochemical role in improving the yield and quality of cucumber. Earlier studies have assessed the method of applying NPs, such as through direct soil application [34], hydroponic [35], seed priming [36], and seed coating [37], and concluded that foliar application is most effective for plant growth and biofortification under both normal and abiotic stress conditions [26,38]. It has been noted that foliar application of NPs increased the concentration of the respective micronutrients in barley, rice and wheat, which led to an enhanced yield and quality [19,39,40]. Foliar-applied NPs enter the cells through the leaf through cuticle or stomata, and are then transported into the seeds through apoplastic and symplastic pathways, into the vascular system [41,42]. Our results concerning the increase in Zn and Fe content in the fruits and seeds of cucumbers confirmed the efficacy of low concentration NPs in bio-fortification through foliar spray. The better absorption of NPs, *vis-a-vis* the application of higher doses of micronutrients, is facilitated because of their physical properties, for example, their minute size (less than 100 nm), higher surface area, reactivity, solubility, penetration capacity, and surface/volume ratio [18], thereby permitting their controlled and targeted delivery [15,43].

The foliar application of ZnNPs and FeNPs, followed by their application as fertilizer, boosted the plant growth in terms of vine length, foliage, number of fruits per plant, fruit size and weight compared to the non-treated control group. In addition to accumulating photosynthate as an active sink, the cucumber fruits also participate in the production of photosynthate [44]. The larger fruit size, and thus larger area of exocarp, could explain the increase in the fruit weight on the treated plants. Further investigation on the net photosynthetic rates, in an open field compared to a protected environment, will delineate a higher gain in most of the plant growth parameters in the open field environment compared to a protected environment

Generally, seed yield in cucumber is a function of the number of fruits per unit area, the number of seeds per fruit and the average seed weight of individual seeds [45]. Improved plant growth, total chlorophyll content and fruit attributes in NPs-treated plants contributed to the increase in seed yield components, such as the total filled seeds per fruit, 1000-seed weight and seed yield per hectare. The role of Zn in chlorophyll production, maintaining structural stability of cell membranes, protein synthesis, cell elongation, tolerance to environmental stresses and as co-factor for various enzymes has been reported in many studies [33,46,47]. The structural alterations also enhance leaf area, net carbon dioxide assimilation, sub-stomata CO_2_ concentration and Fv/Fm ratio [21,37]. In addition, iron is essential for chlorophyll synthesis, photosynthesis and various enzyme systems, such as antioxidants, in plants [48]. Both the micronutrients help in the development and function of reproductive structures, such as gametophyte development, pollen formation, pollen germination, pollen tube growth, fertilization, the development of embryo and seed, as has been reported in various studies [49,50,51]. The higher number of filled seeds and the increase in 1000-seed weight in treated seeds reiterates the improved reproductive success, accompanied by the higher accumulation of seed storage materials, such as TSS, TSP, starch, oil and mineral contents in the treated plants. Zinc and iron are known to regulate various enzymes of protein and carbohydrate synthesis and energy metabolism [52,53]. The activity of soluble starch synthase involved in the synthesis of starch, and also in the number and size of starch grains in *Phaseolus vulgaris*, is controlled by Zn [54,55].

In the present study, the seeds obtained from the NPs-treated plants showed higher seed germination, seedling length, seedling dry weight and seed vigor. The larger and bolder seeds obtained from the NP-treated plants, as a result of better photo assimilate and Zn/Fe supply to the developing seed, improved the seed performance during germination and seedling establishment. The relationship between seed size, germination and seedling vigor in cucumber has been derived in experiments conducted by Upadhyaya et al. [56] and Vaughton and Ramsey [57]. The higher germination in NPs-treated seeds could be due to the increased and rapid water uptake and higher activities of hydrolytic enzymes, i.e., β-amylase and protease. These increased hydrolytic enzymes may provide more oligosaccharides and proteins to germinating seeds [58,59]. Generally, zinc and iron act as starter nutrients during seed germination, as ferritin that is stored in the vacuoles serves as an iron store for the seedling upon germination and seedling growth [60]. On the other hand, Zn is a co-factor of tryptophan, which is a precursor of indole acetic acid and plays an essential role in root formation, growth and primordium development during seedling development [61,62]. These micronutrients are localized in the embryo, endosperm and aleurone layer of the seeds and during seed germination they mobilize into the embryo and scutellum [63,64]. At the cellular level, higher dehydrogenase activity and lower EC values from seed leachates indicated better germinability in the seeds obtained from NP-treated plants. Both of these parameters are considered as biomarkers for seed viability, and seeds with higher metabolic activity are linked to increased dehydrogenase activity and low EC.

The ROS defense system, together with other reactive species and plant hormones, plays an important role in the plant signaling network, related to seed germination, dormancy, seedling establishment, plant growth, plant stress response, cell division and cell death [24]. As Zn and Fe are cofactors of various antioxidant enzymes, they play a key role in maintaining biological redox system in the cells [21,33,46]. Normally, the level of ROS is under the stringent control of antioxidants, which decides the status of the seed during germination, dormancy or ageing. In the present study, regardless of varieties and environment, the content of both ROS (H_2_O_2_ and O_2_^−^) and the activity of antioxidant enzymes (SOD, CAT & POD) was high in seeds from ZnNPs- and FeNPs-treated plants in a concentration dependent manner. Similarly, the activation of antioxidant machinery was observed when FeNPs were applied, hydroponically, onto cucumber seedlings [35]. The increased activity of the antioxidant enzyme helped in the homeostatic control of ROS levels in the cells; this explains the improved seed germination and vigor in the NP-treated seeds in comparison to the seeds supplied with higher amounts of NPs. The level of hydrogen peroxide and superoxide had a promotional effect on the germination characteristics of the treated seeds as they act as secondary messengers and interplay with the hormone signaling pathways to change the transcriptome and proteome of the germinating seed [65]. However, the increase was concentration-dependent, up to a concentration of 300 mg L^−1^, beyond which it became deleterious to the growth attributes, as it reached toxic levels.

Recently, the concentration-dependent effect of NPs for Ni and Al on the plant growth and antioxidant activity of *Nigella arvensis* has been reported [66]. The higher concentration of NPs caused a reduction in the total chlorophyll, dehydrogenases, assimilates supply, higher ROS and water uptake, as well as the higher EC, TSS & TSP from seeds that impaired stomatal conductance, plant water relations and nutritional balance [67,68]. Simultaneously, the toxic effect of lower concentrations of Fe_3_O_4_ NPs was also observed in cucumber [35]. Thus, the standardization of the appropriate concentration of NPs is very important, as a higher concentration may damage the plant tissues and performance. Further studies are required to decipher the mechanism of action of the NPs-mediated increase in seed yield, quality and biofortification in various crops.

## 4. Material and Methods

The present study was conducted with two cucumber varieties, Pusa Barkha and Pusa Uday, during the summer and rainy seasons in 2019, in an open field and under protected conditions (naturally ventilated poly house) at ICAR–Indian Agricultural Research Institute, New Delhi. The soil of the experimental plot (0–30 cm) was analyzed for soil characteristics, such as pH, EC and available Fe and Zn content, by extracting them with a solution of 0.005 M diethylenetriamene pentaacetic acid (DTPA), 0.01 M CaCl_2_·2H_2_O and 0.1 M triethanolamine (TEA) (pH 7.3), and measuring them with atomic absorption spectroscopy (AAS, AAnalyst 400 Perkin Elmer, Waltham, MA, USA), following the method given by Lindsay et al. [69]. The seeds were sown on the ridges with spacing of 2.0 m × 0.5 m in the open field, and 0.6 m × 0.3 m in the protected structure, with three replicates of each treatment in a complete randomized block design (CRBD). A minimum of two hundred plants (100 in each variety) on trellis were maintained under both the environments, and female flowers from each plant were hand pollinated, in the morning between 0700 to 0100 h, and tagged. The fruits were harvested from similar nodes at maturity.

### 4.1. Synthesis and Characterization of Zn- and Fe-NPs

The synthesis and characterization of the ZnO-NPs and Fe_3_O_4_-NPs were conducted at the Department of Engineering and Physical Sciences, Institute of Advanced Research, Gandhinagar, Gujarat, India. The two NPs were synthesized using a slightly modified version of the process outlined by Vallabani et al. [70]. In order to prepare the citrate-coated ZnO-NPs, 2.5 g of ZnSO_4_ were dissolved in 50 mL of MilliQ water yielding citrate-coated Fe_3_O_4_-NPs by preparing a solution of 2.5 g of FeCl_3_·6H_2_O and 1.2 g of FeSO_4_·7H_2_O in 60 mL of MilliQ water. With continuous N_2_ bubbling, both solutions were separately heated at 70 °C for 30 min. With constant stirring, each solution was then heated for a further 2 h at 70 °C, before being cooled to room temperature. The supernatant was collected by centrifuging the solutions at 3000 rpm for 10 min. The supernatant had final concentrations of ZnO-NPs and Fe_3_O_4_-NPs as 1 mg and 2 mg, respectively. A Transmission Electron Microscope (JEM1400 plus, JEOL, Tokyo, Japan) was used to measure the size of the particle dispersions of the NPs. Dynamic Light Scattering was used to calculate the NPs’ Zeta potential (Zetasizer Nano-ZS, ZEN3600, Malvern instruments Ltd., Worcestershire, UK).

### 4.2. Foliar Application of NPs, ZnSO_4_ and FeSO_4_

ZnNPs and FeNPs solutions were prepared in MilliQ water at concentrations of 100, 200 and 300 mg/L. The NPs were prepared by dispersing them in MilliQ water using a mechanical stirrer and 30 min of ultra-sonication to prevent the aggregation of the NPs. ZnSO_4_ and FeSO_4_ solutions at 0.5% concentration were prepared in MilliQ water. All of the NPs and the ZnSO_4_ and FeSO_4_ solutions were mixed with polyoxyethylene sorbitan monolaurate (Tween-20) as a surfactant and sprayed onto the plants three times: one at the vegetative stage (20 DAS), the second at flowering and the third after flowering. A total of nine treatments, including control (without foliar spray), were tested. Three concentrations (100, 200, 300 mg/L), each made up of both of the NPs to observe the effect of a bulk form of micronutrient (0.5% ZnSO_4_ and 0.5% FeSO_4_), were applied through spray.

### 4.3. Plant Growth Attributes

The length of vine and the total number of fruits per vine were measured randomly from five vines per replicate for each treatment. Fruit weight and dimensions were measured on five randomly selected fruits per replicate for each treatment.

The chlorophyll content of the middle leaves, using 100 mg fresh leaf samples and dimethyl sulphoxide (DMSO), was determined by the non-maceration technique [71]. The absorbance was recorded at 645 and 663 nm using a UV-visible spectrophotometer and total chlorophyll was calculated by the following formula [72].

(1) $$\mathrm{Total}\;\mathrm{chlorophyll}\;\mathrm{content}\;\left(\mathrm{mg}/g\right)=\frac{\left[\left(20.2\times {\;A}_{645}\right)+\left(8.02\times {\;A}_{663}\right)\right]}{W\;\times 1000}\times V$$

where A_645_ = Absorbance at 645 nm; A_663_ = Absorbance at 663 nm; V = Volume of solvent; W = weight of plant sample.

### 4.4. Seed Analysis

Seed dimensions were measured on 25 seeds each in three replicates using a digital Vernier Calliper (RS PRO 150 mm Digital Caliper, India). The total soluble sugar (TSS) from each seed was measured by a phenol–sulfuric acid method, described by [73]. The total soluble proteins (TSP) in each seed were measured using Bradford reagent, using the method given by [74]. The TSP was calculated using standard curve plotted with Bovine Serum Albumin (BSA) and expressed as mg g^−1^ DW in the seed. The starch content from each seed was determined using the Anthrone method described by [75]. The starch was determined using standard curve plotted with D-glucose and expressed as mg g^−1^ DW in the seed. Oil estimation from each seed was determined according to the method described by AOAC [76], using a Soxhlet extractor and diethyl ether as solvent. Each seed sample (5 g) was placed for 8–10 h at the solvent’s boiling point in an extractor unit. After extraction, the sample was put in an oven for 3 h at 105 °C and then re-weighed to derive the oil content, using following formula:

(2) $$\%\;\mathrm{Oil}=\left[\frac{\mathrm{Weight}\;\mathrm{of}\;\mathrm{sample}\;\mathrm{before}\;\mathrm{extraction}\;-\mathrm{Weight}\;\mathrm{of}\;\mathrm{sample}\;\mathrm{after}\;\mathrm{extraction}}{\mathrm{Weight}\;\mathrm{of}\;\mathrm{sample}\;\mathrm{before}\;\mathrm{extraction}}\right]\;\times \;100$$

#### 4.4.1. Quantification of Zinc and Iron Contents in Fruits and Seeds

A one-gram sample from three randomly selected fruits and seeds were dried and digested in an acid mixture consisting of nitric acid: sulphuric acid: perchloric acid, in ratio of 3:2:1. Quantification was performed using an atomic absorption spectrophotometer (AAS, AAnalyst 400 Perkin Elmer, Waltham, MA, USA) and the Zn and Fe contents in the fruits and seeds were expressed in mg kg^−1^ DW.

#### 4.4.2. Seed Germination and Vigor Assessment

The germination test, using the between paper (BP) method and three replicates of fifty seeds from the pure seed fraction, was conducted as per ISTA [77]. The seed vigor indices were computed, following Abdul-Baki and Anderson [78], and using the following formula:

Seed vigor index I = Germination (%) × Total seedling length (cm)(3)

Seed vigor index II = Germination (%) × Seedling dry weight (mg)(4)

To measure the electrical conductivity (EC), ten randomly drawn seeds from each replicate were weighed, soaked in 30 mL MilliQ water and allowed to stand at 25 °C for 24 h. The EC from seed leachates was measured using a digital conductivity meter (CM 183, Elico, India) and calculated using following formula [79]:

(5) $$\mathrm{EC}\;\left({µS\;\mathrm{cm}}^{-1}g^{-1}\right)=\frac{\mathrm{Conductivity}\;\mathrm{reading}\;-\;\mathrm{Back}\;\mathrm{ground}\;\mathrm{reading}}{\mathrm{Weight}\;\mathrm{of}\;\mathrm{seeds}\;\left(g\right)}$$

The dehydrogenases activity from the seeds was estimated following the method given by Kittock and Law [80], using 1% 2,3,5-triphenyl tetrazolium chloride (Tz) solution as a staining agent. The intensity of color was read at 480 nm using a spectrophotometer and methyl cellosolve was used as control.

### 4.5. Seed Physiological Assays

#### 4.5.1. Water Uptake by Seed

The water uptake by the seeds during imbibition or germination, from 0 to 84 h, was determined in three replicates; each replicate had 25 seeds. The seeds were removed and weighed at 2 h intervals. Changes in weight, due to imbibition, were expressed as the amount of water absorbed per seed dry weight, calculated by the following formula:

(6) $$\mathrm{Water}\;\mathrm{uptake}\%=\left[\frac{\mathrm{Fresh}\;\mathrm{weight}\;\mathrm{of}\;\mathrm{seed}\;-\;\mathrm{Dry}\;\mathrm{weight}\;\mathrm{of}\;\mathrm{seed}}{\mathrm{Dry}\;\mathrm{weight}\;\mathrm{of}\;\mathrm{seed}}\right]\times 100$$

#### 4.5.2. Assay of Hydrolytic Enzymes from Germinating Seeds

For β-amylase activity, germinating seeds (0.5 g) at different sampling times (0 to 90 h) were homogenized with ice-cold 16 mM sodium acetate buffer (pH 4.8). The homogenate was centrifuged at 12,000× *g* for 15 min and supernatant was used for β-amylase assay [81]. The enzyme activity was expressed as mg maltose produced mg^−1^ protein.

For protease activity, germinating seed samples (0.5 g) were crushed in 0.2 M-phosphate buffer (pH 7.6) in a chilled mortar and pestle placed in an ice bucket. The mixture was filtered and centrifuged at 12,000× *g* for 30 min at 4 °C. The reaction mixture contained 100 µL of aliquot and 200 µL of phosphate buffer, and reaction was initiated by adding 1% casein as substrate. After incubating the tubes at 50 °C for 2 h, the reaction was terminated by the addition of 5% Trichloroacetic acid (TCA) solution [82]. The mixture was centrifuged at 15,000× *g* for 10 min at 4 °C and protein contents of supernatant were estimated by Bradford [74] method. The absorbance was read at 595 nm and the enzyme activity was expressed as mg protein hydrolyzed g^−1^ FW.

#### 4.5.3. Measurement of Superoxide Radical Content

The superoxide anion (O_2_^−^) production was measured by the ability to reduce nitro blue-tetrazolium (NBT) [83]. The seedling (0.5 g) was homogenized in liquid nitrogen and sodium phosphate buffer, containing 1 mM diethyl dithiocarbamate, to inhibit the superoxide dismutase activity. The absorbance of the reaction mixture (3 mL), containing 2.85 mL of phosphate buffer (0.2 M; pH 7.2 with 1 mM diethyl dithiocarbamate), 100 μL of 0.75 mM NBT and 50 μL of supernatant, was recorded at 540 nm and expressed as ΔA_540_ min^−1^ g^−1^ FW.

The hydrogen peroxide (H_2_O_2_) content was determined by measuring the absorbance of titanium–hydro-peroxide complex [84]. One gram of seedling was crushed in 10 mL cooled acetone and filtered using Whatman No. 1 filter paper, followed by the addition of 4 mL of titanium reagent (titanium dioxide and potassium sulphate digested with concentrated H_2_SO_4_) and 5 mL of ammonium solution to precipitate the titanium-hydro-peroxide complex. The complex was dissolved in 2 M H_2_SO_4,_ and the absorbance of supernatant was read at 415 nm against 2 M H_2_SO_4_ as the blank.

#### 4.5.4. Antioxidant Enzyme Activity

The superoxide dismutase activity (SOD) was assayed by monitoring the 50% inhibition of photochemical reduction of nitro blue tetrazolium (NBT) [85]. The germinating seeds (0.2 g) were homogenized in 2 mL of 100 mM potassium phosphate buffer (pH 7.0), containing 1 mM Ethylenediaminetetraacetic acid (EDTA) and 1% polyvinyl pyrrolidone (PVP). The homogenate was centrifuged at 15,000× *g* at 4 °C for 30 min and supernatant was used as enzyme extract. The 3 mL reaction mixture contained 50 mM potassium phosphate buffer (pH 7.8), 13 mM methionine, 25 mM NBT, 2 µM riboflavin, 0.1 mM EDTA, 50 mM sodium carbonate and 0.1 mL enzyme extract. The test tubes were illuminated for 15 min at a light intensity of 3600 lux, thereafter reaction was stopped in dark. The absorbance at 560 nm was recorded against blank.

The catalase (CAT) activity was measured by quantifying the residual H_2_O_2_ in the reaction mixture [86]. The reaction mixture comprised of 100 µL enzyme extract, 1.5 mL phosphate buffer (50 mM, pH 7.0), 100 µL H_2_O_2_ (10 mM) and 1.3 mL MilliQ water. Following the addition of H_2_O_2_ to the reaction mixture, a decrease in the absorbance was noted at 240 nm for one min at an interval of 30 s. The CAT activity was expressed as µmol min^−1^ g^−1^ FW.

The peroxidase (POD) activity was assayed by measuring the formation of tetraguaiacol (∈ = 26.6 mM^−1^ cm^−1^) from guaiacol [87]. One g of seed was ground with 0.5 mL of phosphate buffer (50 mM, pH 7.0) containing 1% PVP and 1 mM EDTA. After the centrifugation of the homogenate, the supernatant was used as an enzyme extract. The 3.0 mL reaction mixture comprised 1.5 mL phosphate buffer (50 mM, pH 7.0), 0.96 mL 16 mM guaiacol, 120 µL 2 mM H_2_O_2_, 100 µL enzyme extract and 0.32 mL MilliQ water. Changes in absorbance up to 3 min was measured at 470 nm and the POD activity was expressed as µmol tetra-guaiacol formed min^−1^ g^−1^ FW.

### 4.6. Statistical Analyses

The data obtained from the experiments are expressed as mean ± S.E.M of three replicates. Statistically significant differences among treatments, varieties and environments were determined following analysis of variance (ANOVA) and the treatment means were compared with a Tukey test (*p* ≤ 0.05) using SAS, version 9.3. The data recorded as percentage were transformed to the respective angular (arc sin) values, to normalize the data, before subjecting it to statistical analyses.

## 5. Conclusions

Foliar spraying of ZnNPs at 300 mg L^−1^ and FeNPs at 200 mg L^−1^ at three growth stages improved the vine length, number of fruits per plant, total leaf chlorophyll content, fruit attributes (dimension and weight), seed accumulates (TSS, TSP, starch and oil content) and, finally, seed yield attributes (number of seeds per fruit, 1000 seed weight, seed yield per hectare), compared to the untreated plants and the plants treated with higher doses of NPs and Fe and Zn solutions. The accumulation of Zn and Fe content in fruits and seeds, upon the foliar application of NPs, showed the potential benefit of this application for the purpose of biofortification. This method is more advantageous than soil application as it can help increase their contents in the fruits and seeds without a simultaneous residual effect in the soil, that may cause environmental pollution. As the seeds obtained from the NPs-treated plants showed enhanced seed germination and vigour, it helps in boosting the seed quality through improving germination characteristics. Understanding the physiochemical effect of foliar-applied ZnNPs and FeNPs on cucumber will pave the way for its prospective application in the future, to improve seed yield and quality.

## Figures and Tables

**Table 1:** Soil properties at open and protected experimental sites.

Site	Texture	pH	EC (dS m^−1^)	DTPA-Extractable Fe (mg kg^−1^)	DTPA-Extractable Zn (mg kg^−1^)
Open field	Sandy loam	7.5–7.8	0.40–0.90	8.5–15.7	0.8–2.2
Protected	Sandy loam	7.6–7.9	0.42–0.88	8.9–15.5	0.9–2.1

## Data Availability

Data are available from the authors upon request.

## Supplementary Materials

The following supporting information can be downloaded at: https://www.mdpi.com/article/10.3390/plants11233211/s1, Figure S1: Effects of foliar application of Zn & Fe (nano & bulk) particles on speed of germination, AUC, mean germination time and t50 in cucumber seed grown under different environments; Table S1: Effects of foliar application of Zn and Fe (NPs and bulk) on seed dimensions and number of filled seeds in cucumber under open field (E1) and protected environment (E2).
