# Supplementary material for: Impact of Foliar Application of ZnO and Fe3O4 Nanoparticles on Seed Yield and Physio-Biochemical Parameters of Cucumber (Cucumis sativus L.) Seed under Open Field and Protected Environment vis a vis during Seed Germination
Source: Plants (Basel). 2022 Nov 23;11(23):3211. doi: 10.3390/plants11233211 (PMC9738616; doi:10.3390/plants11233211)
Supplement: Supplementary file 1 [file plants-11-03211-s001.zip › plants-2004592-supplementary.pdf]

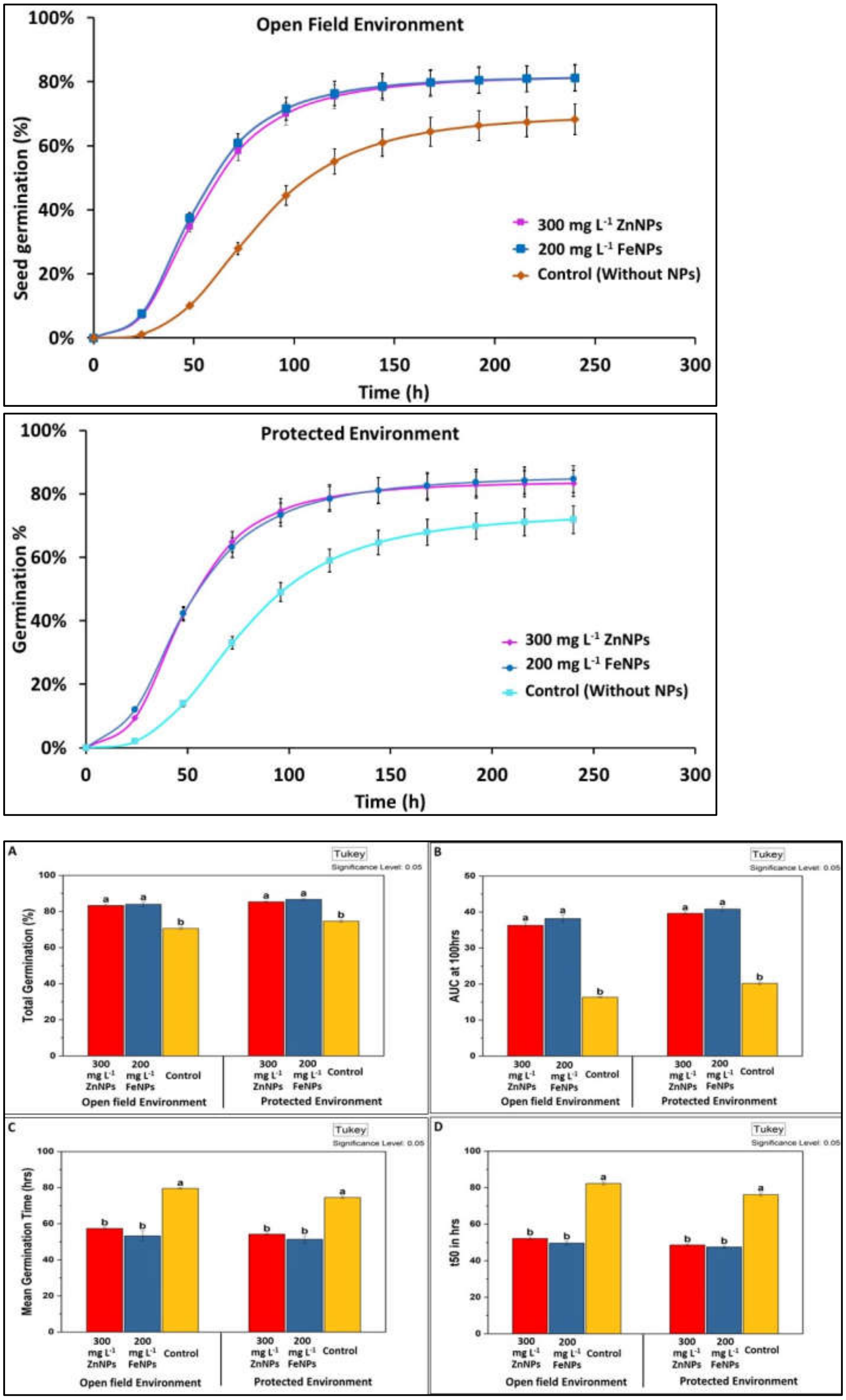

Figure S1. Effects of foliar application of Zn & Fe (nano & bulk) particles on speed of germination, AUC, mean germination time and t50 in cucumber seed grown under different environments. (Data represent the mean values ± SE of two varieties from two seasons using three replicates).

Table S1. Effects of foliar application of Zn and Fe (NPs and bulk) on seed dimensions and number of filled seeds in cucumber under open field (E1) and protected environment (E2)

Treatment	Seed Length (mm)		Seed Breadth (mm)		Total Number of Filled Seed per Fruit	
	E1	E2	E1	E2	E1	E2
100mg L^{-1} ZnNPs	10.39 ± 0.01ab	11.03 ± 0.01ab	3.90 ± 0.01a	4.08 ± 0.01a	297.5 ± 9.5b	326.8 ± 10.2b
200mg L^{-1} ZnNPs	10.42 ± 0.02a	11.09 ± 0.01a	3.96 ± 0.01a	4.16 ± 0.01a	307.5 ± 10.1ab	335.0 ± 8.8ab
300mg L^{-1} ZnNPs	10.51 ± 0.01a	11.15 ± 0.02a	4.02 ± 0.01a	4.17 ± 0.01a	347.5 ± 8.6a	374.8 ± 13.4a
100mg L^{-1} FeNPs	10.37 ± 0.02ab	11.05 ± 0.01a	3.99 ± 0.01a	4.11 ± 0.01a	301.8 ± 12.3b	328.0 ± 13.0b
200mg L^{-1} FeNPs	10.50 ± 0.01a	11.13 ± 0.01a	4.01 ± 0.01a	4.16 ± 0.01a	336.5 ± 11.5a	365.8 ± 12.4a
300mg L^{-1} FeNPs	10.41 ± 0.01a	11.09 ± 0.01a	3.88 ± 0.01a	4.06 ± 0.01a	310.3 ± 10.3ab	339.5 ± 11.9ab
0.5% $ZnSO_4$	10.33 ± 0.01b	11.00 ± 0.02ab	3.89 ± 0.01a	4.01 ± 0.01a	286.5 ± 13.4b	315.3 ± 11.4b
0.5% $FeSO_4$	10.31 ± 0.01b	11.01 ± 0.01ab	3.88 ± 0.01a	4.00 ± 0.01a	280.8 ± 11.7bc	310.3 ± 10.6bc
Control	10.30 ± 0.02b	10.96 ± 0.01b	3.81 ± 0.01a	3.99 ± 0.01a	254.5 ± 10.7c	288.0 ± 10.0c

Values in the table are mean of two varieties from two seasons using three replications ± standard error (SE).
